# Pathological Provings

**Published:** 1891-11

**Authors:** 


### HOMŒOPATHIC PHYSICIAN,

A MONTHLY JOURNAL OF

HOMŒOPATHIC MATERIA MEDICA AND CLINICAL MEDICINE.

"If our school ever gives up the strict inductive method of Hahnemann, we are lost, and deserve only to be mentioned as a caricature in the history of medicine."—CONSTANTINE HERING.

Vol. XI. NOVEMBER, 1891. No. 11.

### EDITORIAL.

PATHOLOGICAL PROVINGS.—In the monthly *Homœopathic Review* for September 1st, appears a paper by Mr. C. Knox Shaw, M. R. C. S., entitled "Observations on the Action of Iodide of Potassium in Tertiary Syphilis," which paper was read before the British Homœopathic Congress at its meeting last July, in London.

In this article the writer refers to the "universal acceptance of the curative power of the Iodide of Potassium in tertiary syphilis," the power of the drug in massive doses to remove "gummatous deposits."

In a well-prepared effort he endeavors to show that this action of the Iodide is in obedience to the homœopathic law. He is unable to find in the homœopathic provings in the *Cyclopedia of Drug Pathogenesy* any symptoms that would indicate it to be a similar; he, therefore, has recourse to the writings of the old school of medicine, from which he extracts evidence of the pathogenetic powers of the Iodide in producing eruptions resembling syphilitic eruptions.

The whole tenor of the article, is that, in order to cure these pathological conditions, the remedy used must have been proved until it actually produced such organic pathological states. This too seems to have been the impression of several members of the Congress who discussed the paper, as appears from the report of their remarks following as printed in the same journal.

Here is a signal error in the conception of Homœopathy by these distinguished speakers.

If a drug must be able to produce the full organic change for which it is given as a curative agent, then the development of our materia medica must come to a standstill; for who will endure the necessary sufferings and risk of death in order to push a certain drug-action so far? Even if such persons be found, and they do experience a tumor or other pathological product as a result of systematic proving, then it follows that for every case of that particular kind the same remedy must be given, and it must be followed by a cure. Well, then, that makes it a *specific*. But it is an axiom in our school that there is no such thing as a specific. Yet, according to the testimony of these gentlemen, Iodide of Potassium *is* a specific for tertiary syphilis. Then it must cure every case. Does it? The history of the use of Quinine in the treatment of intermittent fever with the numerous disappointments therefrom is a type of the action of drugs in general, and shows how much expectation may be entertained that Iodide of Potassium will cure every case of syphilitic gummata.

To return to our original line of thought, suppose we find two remedies, both of which have as a part of their pathogenesis the same organic change; which one shall we give as the curative? Manifestly, we shall be obliged to try them one after the other. Suppose we have three, four, or more remedies, all of which produce the same pathological state. Then we shall be obliged to try them all one after the other. This brings us back then to rank empiricism, and all the routine, of the dominant school of medicine. Yet Homœopathy is claimed to have rescued us from this darkness. Here then is a *reductio ad absurdum.*

There can be no escape from such conclusion so long as we persist in looking at disease as an entity with a never-changing aspect. So long as we treat *diseases* rather than sick conditions.

The provings of Homœopathy did not produce diseases, but the *semblance* of them. These provings produced *sick conditions.* They brought out the individualities of people. The symptoms by which we are enabled to make a prescription are not the symptoms which are characteristic of the disease and therefore common to every patient having that disease; if it were so, we should be giving the same remedy to every case and this would be specific practice. On the contrary we must be led to our prescription by those symptoms which are individual to that patient; symptoms which we will not see repeated in the next case of that disease which we may have to treat. A failure to find such individual symptoms, and to fit a remedy to them will be followed by a failure to cure.

Therefore, it may be said with confidence that when the gentlemen of the British Homœopathic Congress endeavor to find a remedy for pathological changes in some drug that in massive doses has produced such pathological changes, they are fumbling in the dark, and the light that guides them is not the star of homœopathic truth but the will-o'-the wisp of error.

